# A Cross-Sectional Study of the A1 Phenotype in the Colombian Caribbean: A Shadow Behind Pulmonary Embolism

**DOI:** 10.7759/cureus.68520

**Published:** 2024-09-03

**Authors:** Luis Fernando Saldarriaga Osuna, Felipe A Muñoz Rossi, Adrián Diaz, Melisa Núñez De Larosa, Edgar Dario Mosquera López, Maria Clara Mejia Fajardo, Alba del Pilar Rodriguez Cortes, Jonathan H Coronel Arauz, Antonio J Reche Martinez, Natividad Rico Rios

**Affiliations:** 1 General Medicine, Universidad Nacional de Colombia, Bogotá, COL; 2 Internal Medicine, Universidad Nacional de Colombia, Bogotá, COL; 3 Hematology, IMED Hospitales, Alicante, ESP; 4 Microbiology, Universidad Nacional de Colombia, Bogotá, COL; 5 General Medicine, Central University of Ecuador, Quito, ECU; 6 General Medicine, Cooperative University of Colombia, Medellín, COL; 7 Obstetrics and Gynecology, Hospital Materno Infantil José Domingo De Obaldía, David, PAN; 8 Laboratory Medicine, Puerto Real University Hospital, Cadiz, ESP; 9 Laboratory Medicine, Puerta del Mar University Hospital, Cadiz, ESP

**Keywords:** blood abo group, deep venous thrombosis (dvt), pulomary embolism, venous thromboembolism (vte), venous thrombosis (ijv)

## Abstract

Introduction: Venous thromboembolic disease (VTE) is an episodic condition of multifactorial origin, commonly manifesting as deep vein thrombosis (DVT) and pulmonary embolism (PE). VTE is a major cause of morbidity and mortality. As an acute condition, it has the potential for recurrence and is associated with major consequences; this disease poses significant challenges to the healthcare system. VTE is a widespread concern in developed and developing countries; therefore, it is not limited to specific regions or populations.

Objectives: To evaluate the risk factors associated with unprovoked PE in patients in a hospital center in Sincelejo, Colombia.

Methods: This is an observational, analytical cross-sectional study utilizing retrospective data. From 2010 to 2023, we reviewed 126 medical records of patients who experienced their first unprovoked VTE events and met the inclusion criteria. We performed data analysis using R software version 3.5.1.

Results: Of the patients, 36.5% (n = 46) were women; 63.5% (n = 80) were men, with a mean age of 62.22 years (SD = 10.62). About 53% of women presented with PE, compared to 47% of men. The coagulation factor VIII acted as a PE risk factor (p = 0.098). The best model to predict PE development obtained an Akaike information criterion (AIC) of 176.67, indicating that the A1 positive phenotype is the risk factor with the highest prediction for PE occurrence.

Conclusions: High levels of coagulation factor VIII and an A1-positive phenotype are risk factors that may increase PE development. These findings suggest the need for preventive strategies in this risk setting to reduce the incidence and recurrence of PE.

## Introduction

Thrombosis is the formation of a clot inside a blood vessel, which can partially or completely obstruct blood flow. Thrombi can be located in arteries, veins, or capillaries, presenting differences in their structure depending on the location. Venous thromboembolism (VTE) is an episodic condition of multifactorial origin with environmental and genetic causes that, to be present, requires the concurrence of several risk factors in the same individual, which is not always evident. The common manifestations of VTE are deep vein thrombosis (DVT) affecting the extremities and pulmonary embolism (PE), which often occurs as a complication of DVT. Approximately 60% of all cases of VTE present only as DVT, while the remaining 40% present as PE with or without DVT [1].

VTE remains a major cause of morbidity and mortality, annually affecting nearly one million people in the United States and more than 700,000 people in Europe [2]. In the United States, the mortality rate of PE was 2.81 per 100,000 people in 2019, affecting women in greater proportion (54.7%) [3]. Of cadavers admitted for sudden death to the National Institute of Legal Medicine and Forensic Sciences in Medellin (Colombia), of 46097 medico-legal necropsies performed during the years 2010-2020, 164 cases (0.36%) presented PE and 52.4% presented DVT of lower limbs; the mean age was 57 years (SD 19.3), and 51.2% of those affected were women [4].

The Scientific and Standardization Committee (SSC) of the International Society on Thrombosis and Hemostasis (ISTH) categorizes environmentally caused episodes of VTE into three categories: those caused by a transient risk factor, those caused by a persistent risk factor, and those unprovoked [5]. VTE is highly heritable, with 50% to 60% of the variation in incidence attributable to gene effects, with a multifactorial, non-Mendelian pattern of inheritance [6].

Genetic risk factors involve mutations in the coagulation system that are responsible for inherited hypercoagulable states. In the general population, seven genetic risk factors have been established; four of them include variants in coagulation factor V (factor V Leiden mutation, protein S defects, protein C alteration, and antithrombin mutation), and the others correspond to alteration of the prothrombin gene, fibrinogen gamma, and non-O blood group [7]. There is evidence of an association between ABO blood types and VTE development, partly due to the genetic influence of the ABO locus on plasma levels of factor VIII/Von Willebrand Factor (VWF), with higher levels found in almost 25% of people in non-O blood group 0 [8].

VTE is a chronic disease with an episodic recurrence risk, and in the absence of long-term anticoagulation, approximately 30% of patients develop recurrence within the next 10 years [9]. This makes it necessary to intensify measures for early identification of risk factors to prevent recurrence of the event, avoiding sequelae and death. This study aims to assess the risk factors linked to PE behavior, which is not due to environmental factors, in patients receiving treatment at a Córdoba and Sucre (Colombia) referral hospital center. This evaluation enables personalized treatment decisions to prevent disease recurrence.

## Materials and methods

We conducted an observational, cross-sectional, analytical study with retrospective data. The study evaluated the risk factors associated with unprovoked PE in patients who attended the outpatient hematology department in Sincelejo (Colombia).

The study included 126 patients over 18 years of age, attended from 2010 to 2023 in the outpatient service of hematology, with a diagnosis of a first unprovoked venous thromboembolic episode. Patients under 18 years of age and those who had experienced a recurrence of venous thrombotic events were excluded from the study. Also excluded were individuals who presented with VTE due to: pregnancy or puerperium, surgical interventions in the last three months requiring general anesthesia for at least 30 minutes, active cancer, prolonged immobilization (at least three days) in the previous three months for any cause, oral contraceptive use in the last three months, myeloproliferative syndromes, nephrotic syndrome, hematological disorders with plasma hyperviscosity, and chronic inflammatory disorders (rheumatoid arthritis, inflammatory bowel disease, and others).

The information analyzed was obtained from secondary documentary sources, from the medical records of the patients who attended the hematology outpatient clinic from 2010 to 2023. The essential instrument for the collection of demographics, clinical, and laboratory data was the Microsoft Excel program. This software was used to create a matrix where the study variables were recorded, and once the data collection was completed, it was exported to the statistical program R version 4.3.1.

Statistical analysis

An exploratory analysis of the data was carried out to determine the distribution of absolute and relative frequencies for the study variables using a univariate analysis. A bivariate analysis was performed using the chi-square test to evaluate the statistical significance of the variables studied. Subsequently, a multivariate logistic regression analysis was conducted, selecting the most appropriate prediction model using the Akaike information criterion (AIC) with the lowest value as the selection criterion. For the selected prediction model, accuracy, precision, recall, specificity, and F1-score were calculated. Additionally, the log odds ratios and prevalence ratios (PR) were computed for each independent variable in the chosen model to assess the risk of recurrence of the event of interest.

Ethical considerations

Considering the retrospective nature of the study, the research is classified as low-risk. We did not carry out any interventions that could alter the behaviors or treatments of the participants, thereby eliminating the need to obtain informed consent. Confidentiality of health data was assured, omitting any personal or related information about the medical personnel involved. Approval to conduct this study was obtained from the corresponding ethics committee at the health institution, in strict compliance with good clinical practice regulations.

## Results

In this study, 126 medical records of patients who experienced a first episode of VTE were analyzed. It was found that 36.5% (n = 46) of the patients were female and 63.5% (n = 80) were male, whose mean age was 62.22 years and standard deviation (SD) of 10.62. It was found that 49.2% (n = 62) of the patients presented PE, and 50.8% (n = 64) did not manifest it. Table 1 describes the demographic characteristics and the results of laboratory tests reported in the medical records.

**Table 1:** Sociodemographic characteristics

Variables	n = 126
Age (mean)	62 (95% CI 60.3-64.0)
Sex	
M (%)	80 (63.5%)
F (%)	46 (36.5%)
Blood type	
Blood group 0 (%)	38 (30.2%)
Blood group A (%)	60 (47.6%)
Blood group B (%)	20 (15.9%)
Blood group AB (%)	8 (6.3%)
Venous thromboembolic event	
Pulmonary embolism (%)	62 (49.2%)
Deep venous thrombosis (%)	64 (50.8%)
Prothrombin gene mutation (%)	15 (11.9%)
Leiden factor V (%)	14 (11.1%)
Factor VIII deficiency (%)	24 (14.0%)
Phenotype A1	
Positive (%)	38 (30.1%)
Negative (%)	88 (69.8%)
Homocysteine	
Normal (%)	108 (85.0%)
Elevated (%)	19 (15%)

All laboratory tests associated with VTE were reviewed; however, some of them were not included in the statistical analysis because it was found that there were no alterations in some of these studies in the patients evaluated. The laboratory tests not included in the statistical analysis were antithrombin, protein C, free protein S, lupus anticoagulant, anticardiolipin antibody, anti-beta2-glycoprotein, and protein C resistance.

**Figure 1:**
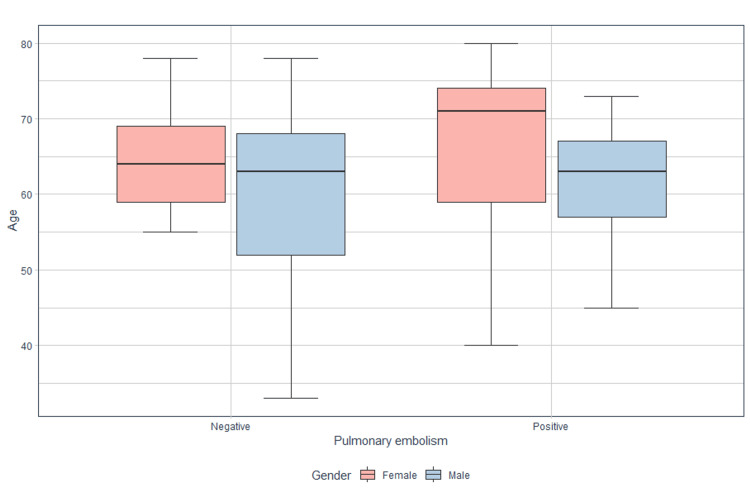
Box plot: pulmonary embolism and age, grouped by gender

The data were subjected to an exploratory analysis, which was carried out using a box plot (Figure 1), in which age was compared as a function of two variables: PE and sex. The mean age for women with PE is 65.5 (95% CI = 59.2-71.8), with a median of 71 years, with extreme values ranging from 40 to 80 years. With comparison to those with negative PE under a mean of 63.6 (95% CI = 60.0-67.2).

In terms of male sex, the mean for males was comparable for both patients with PE 60.9 (95% CI = 56.9-64.8) and those with negative PE 60.9 (95% CI = 57.9-63.9), respectively.

Considering the above, the present analysis suggests that age could be a crucial factor in identifying patients with PE, with differences observed between the sexes. However, these differences are not statistically significant (X^2^ = 1.59, p = 0.207).

**Figure 2:**
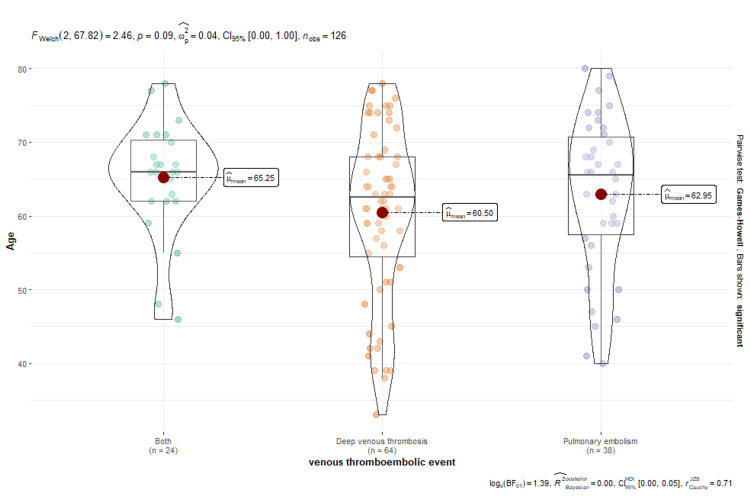
Box plot: venous thromboembolism and age

Figure 2 shows a comparison of means between the different thromboembolic events and the distribution by age, where it is evident that there is no statistically significant difference between the mean ages between the groups of venous thromboembolism (VTE) with a p = 0.09. However, it should be noted that the mean age is higher in the group of patients who presented both events (mean = 65.25).

A chi-square test was done to identify a connection between the PE response variable and each of the specified independent factors. A statistical significance of less than 10% (p < 0.1) with a confidence level of 90% was selected. Table 2 presents the outcomes of the bivariate study, where factor VIII revealed the highest statistical significance (p = 0.098), followed by homocysteine, and last, phenotype A1.

**Table 2:** Bivariate analysis of the independent factors and their relationship with PE df: degrees of freedom; PE: pulmonary embolism

Variables	˜χ^2^	df	p-value
Sex	0.1447	1	0.704
Leiden factor	9.02e-31	1	1.000
Prothrombin gene mutation	0.2048	1	0.651
Elevated factor VIII	2.7358	1	0.098
Non-O blood group	0.0292	1	0.864
Positive phenotype A1	1.7096	1	0.191
Elevated homocysteine	1.9083	1	0.167

Subsequently, a multiple logistic regression analysis was conducted to investigate the influence of independent variables on predicting the occurrence of PE. When the model was applied, incorporating all the evaluated covariates, no statistical significance was observed (Table 3). Consequently, additional logistic regression models were developed using only select independent covariates to identify the model with the highest predictive accuracy for the event of interest based on the AIC. The optimal model yielded an AIC value of 176.67. The independent covariates with the best predictive power for PE recurrence were blood group, homocysteine, and phenotype A1, all demonstrating significant statistical relevance (p < 0.05; 95% CI); the results are detailed in Table 4.

**Table 3:** Multivariate analysis of the independent factors and their relationship with PE. General model PE: pulmonary embolism

Variables	Coefficients	p-value
Sex	-0.1240	0.762
Leiden factor	0.1492	0.804
Prothrombin gene mutation	-0.4476	0.444
Elevated factor VIII	-0.5773	0.276
Non-O blood group	-0.6827	0.176
Positive phenotype A1	0.7970	0.105
Elevated homocysteine	-0.8091	0.148

**Table 4:** Multivariate analysis of the independent factors and their relationship with PE. Selected reduced model OR: odds ratio

Variables	Coefficients	p-value	OR (95% CI)
Non-O blood group	-0.7123	0.1457	0.4905 (0.18-1.26)
Positive phenotype A1	0.9451	0.0373	2.5729 (1.07-6.41)
Elevated homocysteine	-0.8981	0.0958	0.4073 (0.13-1.13)

**Figure 3:**
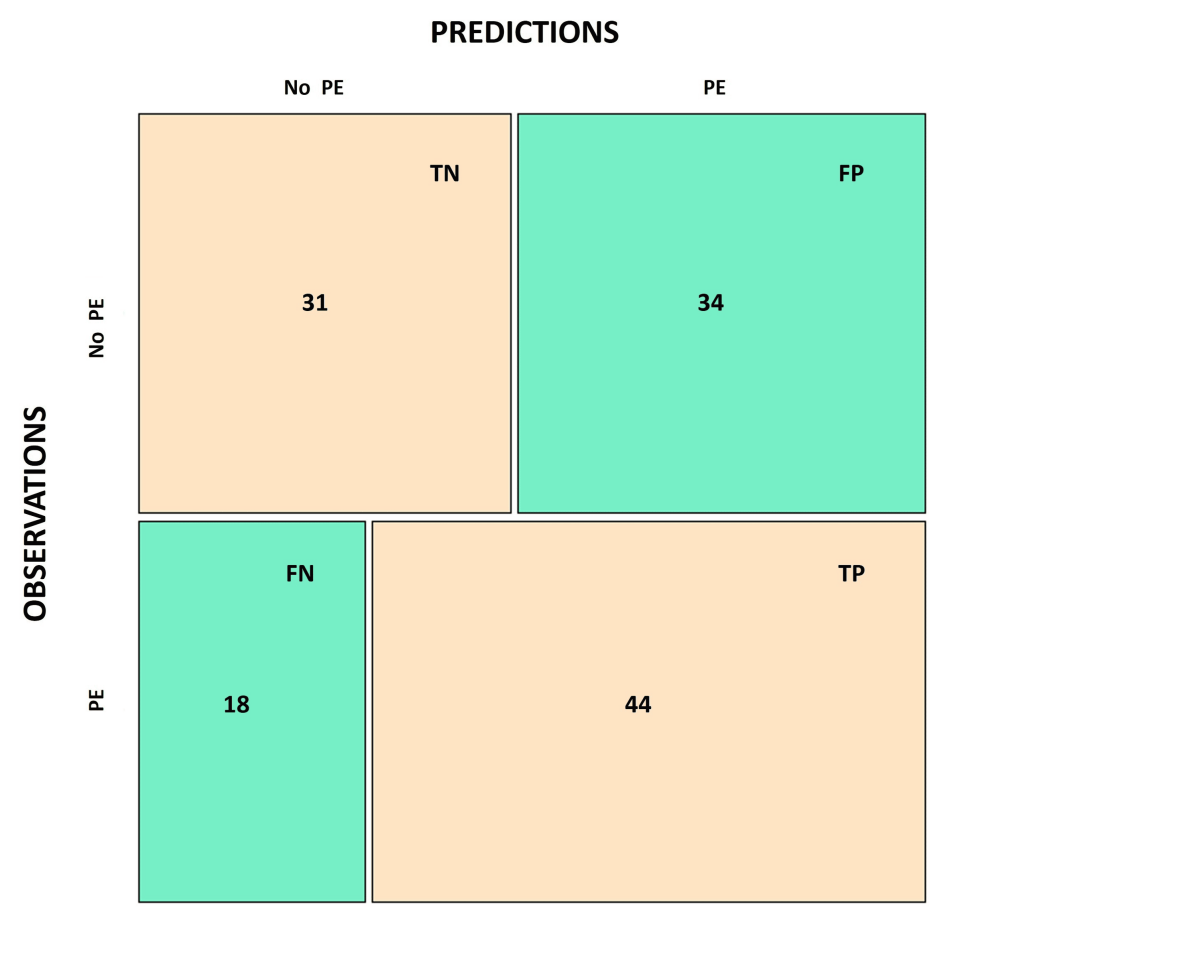
Confusion matrix TP: true positives; TN: true negatives; FP: false positives; FN: false negatives; PE: pulmonary embolism

Based on the selected prediction model, the confusion matrix was constructed (Figure 3), illustrating the number of patients who experienced the event of interest versus those who did not by comparing the model’s predicted values with the actual observed outcomes. In the analysis of the confusion matrix, the selected prediction model was found to have an accuracy of 59.1%, precision of 56%, specificity of 47%, sensitivity (recall) of 71%, and F1-score of 63%.

With the coefficients indicated by the selected prediction model, the PR was calculated for each of the predictor variables since the study was carried out with a cross-sectional time sequence without longitudinal follow-up of the patients. The positive or negative signs of the coefficients indicate the relationship (direct or inverse, respectively) of the predictor variables versus the response variable. The magnitude of the coefficients indicates the logarithmic probabilities by which each of the predictor variables increases or decreases the presence of the event of interest.

Relationship of A1 phenotype and venous thromboembolism

**Figure 4:**
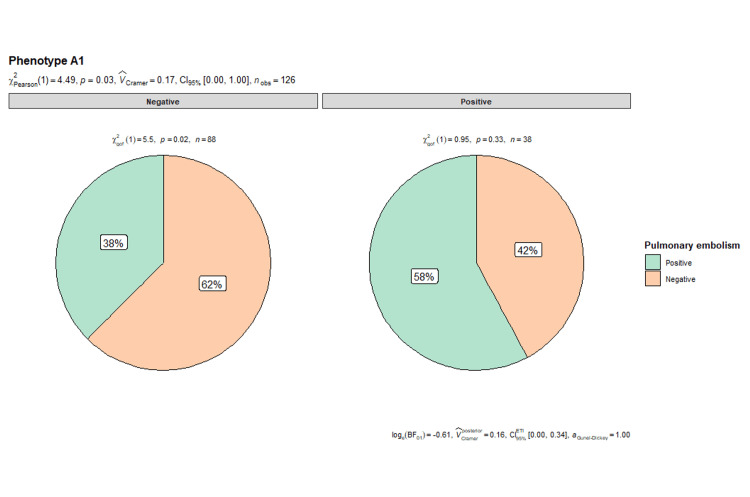
Pie chart: the relationship between pulmonary embolism and the A1 phenotype

In the present investigation, we highlighted the statistically significant relationship between the occurrence of PE and A1 phenotype, with evidence of 58% (22/38) of patients presenting PE having an A1 phenotype present, concerning 38% (33/88) in the group of patients not presenting an A1 phenotype (p = 0.03), with an OR 2.29 (95% CI = 1.06-4.98). As a result, these findings suggest that the A1 phenotype may be an important risk factor in PE development (Figure 4).

**Figure 5:**
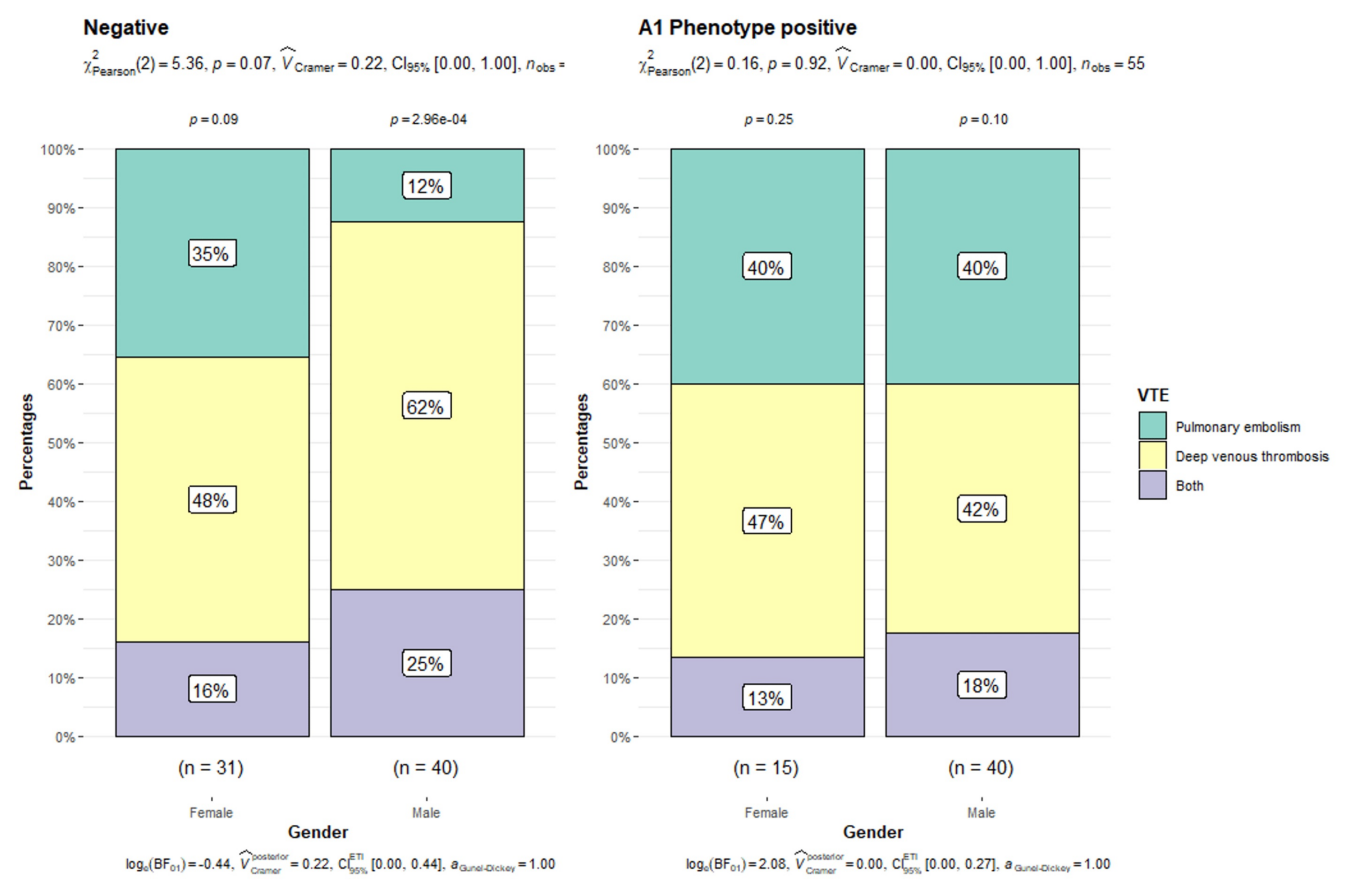
A1 phenotype and gender The X-axis refers to the “gender” of the subjects studied, and the Y-axis represents the “percentages” corresponding to the categories of venous thromboembolism (VTE). The A1 phenotype's presence or absence divides the subjects into two groups.

Regarding gender and A1 phenotype, it is observed that the proportion of PE is higher in patients with A1 phenotype, with no statistically significant differences in gender (X^2^ = 0.16, p = 0.92). In contrast, in the absence of phenotype A1, significant differences are found in the proportion of pulmonary thromboembolism according to gender (X^2^ = 5.36, p = 0.07), with a prevalence of 35% in women (n = 11/31) versus 12% in men (n = 5/40) (Figure 5).

## Discussion

The low index of clinical suspicion when other differential diagnoses are ruled out, the variability in the presentation of the clinical picture, and the low-risk stratification in the absence of obvious triggering factors lead to a delay in the diagnosis of PE [10], despite advances in imaging and laboratory diagnostic techniques. This impacts the high incidence of this condition, representing a health problem with high rates of underdiagnosis. Although its mortality has decreased, in part due to advances in anticoagulation therapies that have reversed recurrences, it remains significant [11,12].

The present study is the first to evaluate the demographic characteristics and risk factors associated with unprovoked PE due to environmental causes in patients from the Colombian Caribbean coast. The importance of the study is to help identify the factors that most predict the development of unprovoked PE and to guide therapeutic decisions to prevent the recurrence of the disease so that the prognosis of affected patients can be improved.

In this study, the mean age of the patients was 62.22 years (SD 10.617), a figure above the average age for studies that evaluated the recurrence of unprovoked VTE in the European population [13,14]. However, it is in the same range as the average age for research conducted in North America [15] and Latin America [16]. The differences in the average age concerning the European studies may be due to the strong predominance of the Caucasian population in that continent, where this type of ethnicity behaves as a clear risk factor for VTE, allowing it to occur at younger ages. On the contrary, the multiethnicity that characterizes the countries of the American continent, with a significant immigrant population and no clear predominance of any race, allows VTE to present at an older age.

The present study shows that men, unlike women, were more affected by unprovoked VTE (65% vs. 35%), a finding that differs from the literature reported in other countries [13-15]. However, for this study, women compared to men presented greater involvement by PE (52% versus 47%), which is related to the information provided by the National Institute of Legal Medicine and Forensic Sciences regarding mortality due to PE in Colombia [4]. The box plot suggests that, for both sexes, patients with PE tend to have higher ages compared to those without PE; it also indicates that women affected by PE have higher ages than the other groups of the population evaluated.

The elevated levels of coagulation factor VIII showed statistical significance in the bivariate analysis performed with the chi-square test. This suggests that this condition behaves as an independent risk factor, which may increase the risk of presenting unprovoked PE. This finding corroborates what has been described in studies carried out in European [13,14], Brazilian [16], and Colombian [17] populations. The other demographic and laboratory variables did not show statistical significance in this type of analysis.

In the multiple logistic regression analysis, the model with the best predictive power for the development of unprovoked PE obtained the lowest AIC index (176.67) compared to the other models evaluated and included the independent covariates: A1 phenotypes, homocysteine, and blood group. When the coefficients of the chosen model are analyzed, it is inferred that the probability of presenting PE is 0.71 times lower in patients with non-O blood type when the A1 phenotype and homocysteine are fixed. Similarly, the probability of presenting PE is 0.95 times higher in patients with the A1 phenotype when the blood group and homocysteine are kept fixed. Finally, when the blood group and A1 phenotype remain fixed, the probability of presenting PE is 0.89 times lower in patients with elevated homocysteine.

When analyzing the PR for each covariate in the chosen prediction model, it was found that phenotype A1 behaves as a risk factor and indicates that the risk of PE recurrence is 2.58 times higher in patients with positive A1 phenotype, compared to those with negative A1 phenotype (p ≤ 0.05); this finding is consistent with the results reported in European and North American population studies, highlighting the role of the A1 phenotype as a risk factor for VTE, suggesting that certain blood group antigens may induce coagulation pathways [18,19]. The covariates blood group and homocysteine are not statistically interpretable since the range of values in which their PR would be expected to be found in the general population crosses the null value at a 95% confidence interval; therefore, their coefficients described above are not statistically interpretable either.

The chosen prediction model presents intermediate specificity, accuracy, F1-score, and precision; however, its pronounced capacity to correctly classify patients with PE with a sensitivity of 71% is highlighted. This sensitivity value suggests that using the variables proposed by the model could be useful as a screening strategy in low-level health institutions, where they can become a simple stratification tool to help select those patients most likely to present unprovoked PE. These patients should benefit from more rigorous and complex evaluations in higher-level health institutions, which allow for confirmation of diagnostic suspicion.

This study has certain limitations. First, the small sample of patients studied limits the external validity of the results reported since the sample studied is restricted to patients who attended the hematology outpatient clinic, which does not represent the general population. The small sample of participants is because the clinical records were selected from a hospital center belonging to a city with a small territorial dimension and low population census, where a very low incidence of idiopathic thromboembolic events is expected to be found. Second, the lack of further information in the medical records, due to the retrospective nature of the data obtained. It cannot be ruled out that the absence of certain records in the clinical histories, comorbidities, other demographic variables (origin, health affiliation regime, social stratum, ethnicity), and some relevant antecedents could have had an impact on the risk of recurrence of unprovoked PE. Third, given the absence of temporal follow-up of patients, longitudinal information is not available, which would help to better understand the role of some transient risk factors in unprovoked PE. Complementary studies are suggested, including a greater number of predictor variables, larger sample sizes, and different methodological designs.

## Conclusions

This study aims to establish a bibliographic basis, evaluating for the first time a statistical prediction model for the development of unprovoked PE based on demographic and laboratory risk factors in a sample of patients from the Colombian Caribbean coast. Older and female patients showed a higher risk of developing PE. In elevated levels, coagulation factor VIII and the presence of a positive A1 phenotype act as independent risk factors that may increase the development of PE. Therefore, these risk factors should be taken into account in the evaluation and treatment of patients who have had an unprovoked thromboembolic event to prevent incidence. However, further research with fewer limitations is required to corroborate the proposed findings.
